# El Niño increases the risk of lower Mississippi River flooding

**DOI:** 10.1038/s41598-017-01919-6

**Published:** 2017-05-11

**Authors:** Samuel E. Munoz, Sylvia G. Dee

**Affiliations:** 1 Department of Geology & Geophysics, Woods Hole Oceanographic Institution, Woods Hole, Massachusetts 02543 USA; 2 Department of Earth, Environmental, and Planetary Sciences, Brown University, Providence, Rhode Island 02912 USA

## Abstract

Mississippi River floods rank among the costliest climate-related disasters in the world. Improving flood predictability, preparedness, and response at seasonal to decadal time-scales requires an understanding of the climatic controls that govern flood occurrence. Linking flood occurrence to persistent modes of climate variability like the El Niño-Southern Oscillation (ENSO) has proven challenging, due in part to the limited number of high-magnitude floods available for study in the instrumental record. To augment the relatively short instrumental record, we use output from the Community Earth System Model (CESM) Last Millennium Ensemble (LME) to investigate the dynamical controls on discharge extremes of the lower Mississippi River. We show that through its regional influence on surface water storage, the warm phase of ENSO preconditions the lower Mississippi River to be vulnerable to flooding. In the 6–12 months preceding a flood, El Niño generates a positive precipitation anomaly over the lower Mississippi basin that gradually builds up soil moisture and reduces the basin’s infiltration capacity, thereby elevating the risk of a major flood during subsequent rainstorms. Our study demonstrates how natural climate variability mediates the formation of extreme floods on one of the world’s principal commercial waterways, adding significant predictive ability to near- and long-term forecasts of flood risk.

## Introduction

The Mississippi River is an economic artery of the United States, and federal efforts to understand, predict, and manage flooding along its course have been underway since the 19^th^ century^1^. On the lower Mississippi (below the Mississippi’s confluence with the Ohio River), flood protection is provided by a system of earthen levees and spillway structures designed to contain discharges exceeding those associated with the largest floods observed during the early 20^th^ century^2^. Floods remain costly despite the protection offered by modern river engineering, with economic damages from flooding in 2011 estimated to be $3.2 billion^3^. Failure of key elements of the current flood control system, which nearly occurred during a major flood in 1973, would be an economic and humanitarian disaster of unprecedented severity^4^. Forecasting flood occurrence over seasonal to decadal time-scales, and thus affirming the viability of these flood protection measures, remains a major challenge – especially in light of the brevity of the instrumental record and the confounding effects of flood control infrastructure on the behavior of fluvial systems^5, 6^, both of which limit our ability to characterize hydrological systems’ sensitivity to climate variability and change^7, 8^.

Improving flood forecasting for the lower Mississippi depends on understanding the links between flood occurrence and the slowly varying, more predictable modes of climate variability that influence hydrological processes over central North America, including the Pacific-North American Pattern (PNA), the Atlantic Multi-Decadal Oscillation (AMO), the North Atlantic Oscillation (NAO), and the El Niño-Southern Oscillation (ENSO)^9–13^. Analyses of historical datasets have identified relationships of varying strength and direction between these modes of climate variability, precipitation, and streamflow over portions of the Mississippi River basin, particularly the Missouri/upper Mississippi^10, 14, 15^ and Ohio River basins^11, 16, 17^. Establishing the dynamical controls on increased flood risk on the lower Mississippi River has proven more challenging due to the multiple interacting controls that govern flood occurrence at the outlet of a continental drainage system^18, 19^, and the limited number of extreme floods available for study during the period of instrumental record.

On major river systems, extreme floods arise from the interaction of atmospheric processes that transport large amounts of oceanic moisture inland^18^ with the properties of the land surface that dictate the rate of surface runoff delivered to the main channel^19^. The relatively slow movement of water through soils relative to atmospheric moisture tranport processes creates lags between river discharge and the state of the climate system^20, 21^. Efforts to understand the causes of floods on the lower Mississippi River have typically focused on the extreme precipitation event(s) in the weeks prior to peak discharge. These rainstorms occur when moist air from the subtropical North Atlantic is concentrated along a frontal zone positioned across the basin, and are linked to the strength and position of the North Atlantic Subtropical High (NASH)^22–24^, a correlate to the phases of the PNA, AMO, and NAO^11, 15, 25^. Considerably less attention has been paid to the climatic controls on antecedent soil moisture – a key element in the development of a flood that evolves gradually but preconditions a basin to be vulnerable to flooding by reducing the infiltration capacity of the land surface^19, 21^ – and its role in generating discharge extremes of the lower Mississippi River.

**Figure 1:**
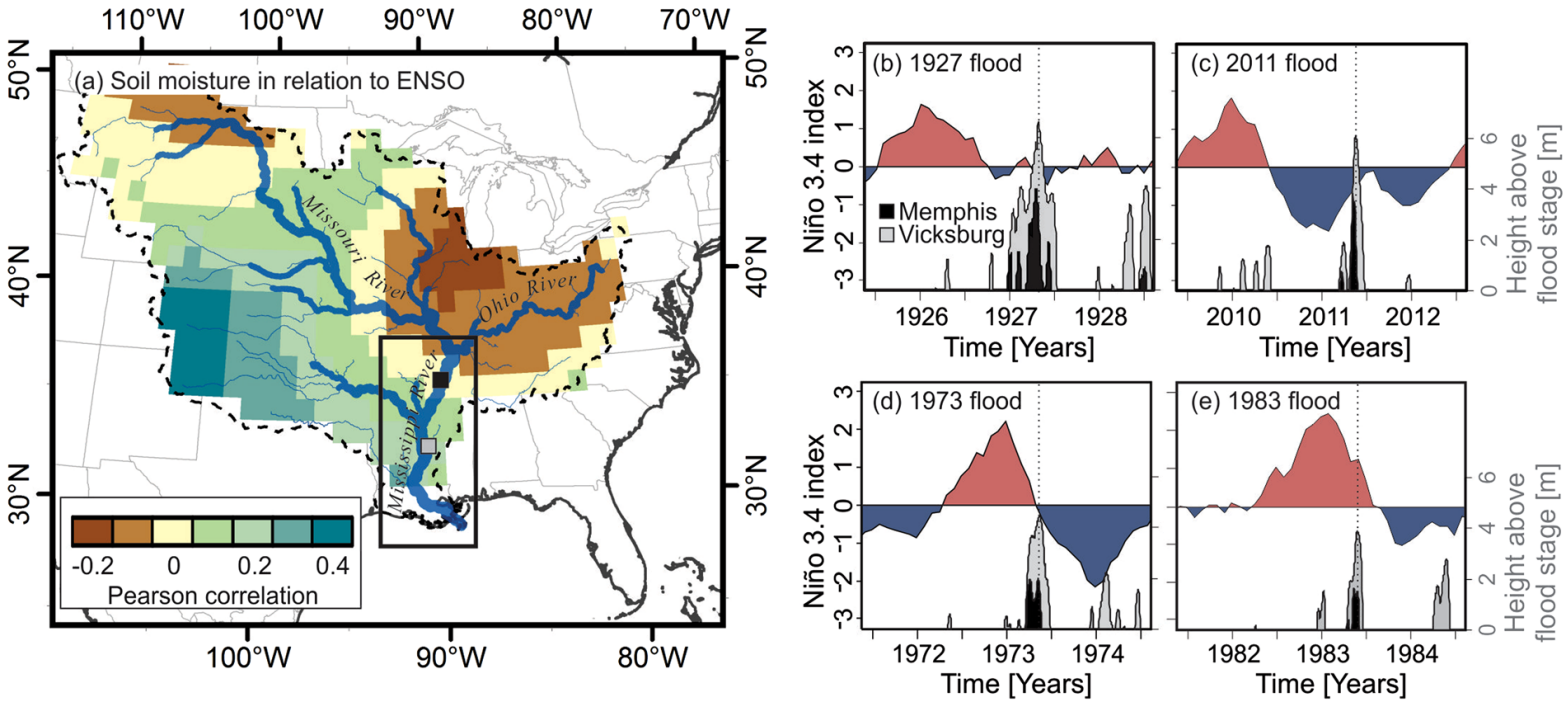
Left panel: The Mississippi River basin and its soil moisture in relation to ENSO, expressed as a Pearson correlation between monthly soil moisture anomalies^46^ and the Niño 3.4 index^35^ from 1870–2014. The locations of the river gauging stations at Memphis, Tennessee (black square) and Vicksburg, Mississippi (grey square) are shown in relation to the lower Mississippi River (box). Right panels: Monthly Niño 3.4 index in relation to daily river stages for the Mississippi River at Memphis and Vicksburg for floods in (**b**) 1927, (**c**) 2011, (**d**) 1973, and (**e**) 1983. River stages are expressed as a height above the flood stage as defined for each gauge^23^. Map in left panel generated in ArcMap v.10.2.2 (http://arcgis.com).

Soil moisture over the lower Mississippi basin is strongly influenced by ENSO^10, 26, 27^ – a dominant mode of climatic variability associated with sea surface temperature anomalies in the eastern equatorial Pacific^28^ – and the two largest historical floods of the lower Mississippi River in the springs of 1927 and 2011 were preceded by El Niño events in the winters of 1925/1926 and 2009/2010, respectively (Fig. 1). More moderate floods, including those in springs of 1973 and 1983, were preceded by El Niño events in the winters of 1972/1973 and 1982/1983, respectively. Of all 14 major Mississippi River floods observed at Vicksburg, Mississippi (defined as peak annual stage >15.24 m)^29^ from 1858–2015, 64% have occurred within a year of an El Niño event (Supplemental Table 1). Through ENSO’s influence on the position and strength of the subtropical and polar jet streams^28^, El Niño events are associated with increased surface water storage over the lower Mississippi River basin^10, 26, 27^ that can persist for months due to the slow release of water stored in soils^20^. Based on these observations, we hypothesized that ENSO modulates lower Mississippi River discharge – and thus flood occurrence – within a year of an El Niño event through its influence on surface water storage.

To investigate the relationship between ENSO and lower Mississippi River floods, we used the Last Millennium Ensemble (LME) of the Community Earth System Model (CESM1)^30^. We evaluated all ‘full-forcing’ ensemble members in the CESM–LME, comprised of 10 realizations for the period A.D. 850–2005 (i.e., 1,155 years for each realization). The CESM–LME includes a coupled river transport module^30^, and simulates a greater number of discharge extremes than are available in the short (i.e., last 100–150 year) instrumental record. From the CESM–LME simulations, we extracted peak annual discharge for the lower Mississippi River basin and sea surface temperatures in the Niño 3.4 region (see Methods for details). We then compared the magnitude and return intervals of peak annual discharges that occurred within 12 months of an El Niño episode with those that did not. We also analyzed the trends in mean monthly soil moisture and precipitation anomalies over the lower Mississippi basin, as well as surface temperature and sea level pressure anomalies across the western hemisphere in relation to extreme floods (defined here as peak annual discharges with an annual exceedance probability ≤1%; i.e., ‘a 100-year flood’).

Prior work validating CESM–LME output has demonstrated that the full-forcing realizations reproduce major modes of observed internal climate variability, including ENSO and its teleconnections^30–33^; we performed additional validation to demonstrate that the mean, variance and seasonality of simulated and observed lower Mississippi River discharge is similar (Supplemental Fig. 1) and that CESM’s soil moisture field in relation to ENSO is comparable to that observed historically (Supplemental Fig. 2). The CESM–LME does not simulate the effects of engineering infrastructure (e.g., artificial levees, dams, and spillways), irrigation, or groundwater extraction on discharge, allowing us to evaluate the climate controls on discharge independently of the effects of most human alterations to the basin that confound analyses of instrumental datasets^2, 5, 6^. Land use is a transient forcing in the CESM–LME that could influence simulated discharge^5–7^, but we found no significant difference in peak annual discharge when we compared the pre- and post-agricultural periods (i.e., AD 850–1800 and AD 1800–2005) in the simulations (unpaired t-test, *t* = 0.2356, df = 2605.3, *p* = 0.8138).

## Results and Discussion

**Figure 2:**
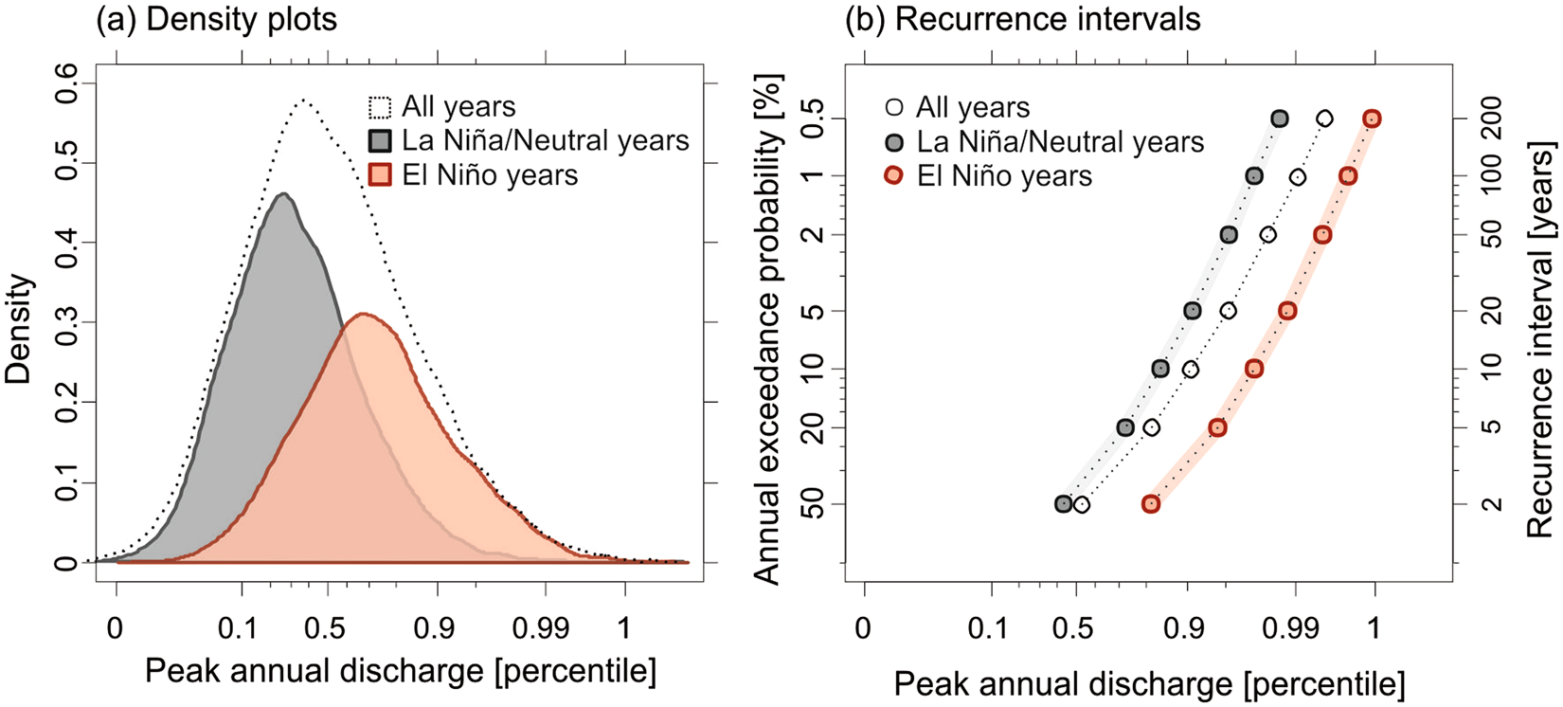
Left panel: Density plots of simulated peak annual discharge (log-transformed) for the lower Mississippi River in all years (dotted line, n = 11,550), La Niña/ENSO-Neutral years (grey, n = 9,191), and El Niño years (red, n = 2,359). Right panel: Recurrence intervals and annual exceedance probabilities in relation to peak annual discharge for all years (dotted line), La Niña/ENSO-Neutral years (grey points), and El Niño years (red points); uncertainties at the 95% confidence level are smaller than the size of the points. Peak annual discharge is plotted as a percentile of all simulated peak annual discharges.

In the CESM–LME, peak annual discharges on the lower Mississippi River that occur within a year after an El Niño event are significantly larger (*p* < 0.001, unpaired t-test on log-transformed data) than those that do not (Fig. 2). Of the 116 extreme floods simulated in the model, 71% occur within a year after an El Niño event. The elevated discharges associated with El Niño years increase the probability that a flood of a given magnitude will occur. For example, the exceedance probability of an extreme flood shifts from 1% (recurrence interval, *t*_r_ = 100 years) in a random year to 3.3 ± 0.6% (*t*_r_ = 30 ± 5 years) following an El Niño event. In other terms, the warm ENSO phase elevates the risk of an extreme flood on the lower Mississippi River by a factor of three when compared to any random year in the model simulation. The risk for the same extreme flood increases by a factor of eight when an El Niño year is compared to an ENSO-neutral or La Niña year. These results imply that ENSO plays a significant role in the lower Mississippi River’s discharge and markedly alters the probability of flood occurrence in a given year.

**Figure 3:**
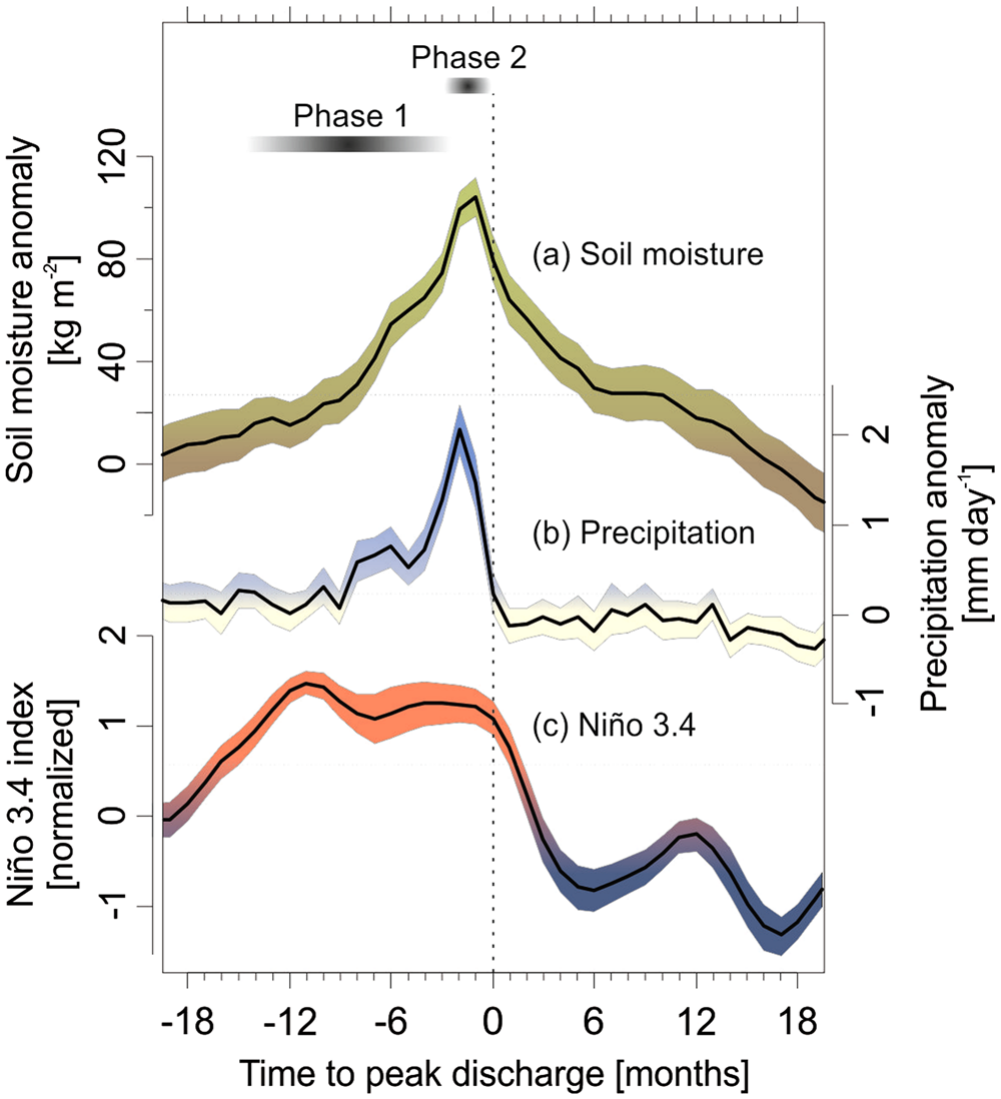
Stacked monthly (**a**) soil moisture anomalies and (**b**) precipitation anomalies over the lower Mississippi basin, and (**c**) the Niño 3.4 index for all simulated extreme floods (n = 116; defined as a peak annual discharge with an exceedance probability ≤1%, i.e., a 100-year flood) in the CESM–LME in relation to the time of peak discharge. The mean (line) and 95% confidence interval (colored silhouette) are shown for each variable. The vertical dotted line represents the month of peak discharge; horizontal lines denote a positive anomaly at the *p* < 0.01 significance level for each variable calculated.

Extreme floods in the CESM–LME tend to be preceded by positive precipitation and soil moisture anomalies over the lower Mississippi River basin (Fig. 3). When all simulated extreme floods (n = 116) are considered together, significant (*p* < 0.01, bootstrapped confidence intervals) positive precipitation and soil moisture anomalies emerge 6–12 months prior to peak discharge, and continue to increase until the month of the flood event. These hydrological anomalies closely follow positive anomalies of the Niño 3.4 index (i.e., El Niño conditions), implying that the hydroclimatic impacts of ENSO on the lower Mississippi River basin observed in instrumental records and other simulations^10, 16, 20, 26, 27^ are realistically simulated in the CESM–LME. At 0–2 months prior to peak discharge, extreme floods in the CESM–LME tend to be preceded by a large influx of precipitation that mirrors the large rainstorm(s) that occur prior to observed floods^22–24^. These large rainstorms are associated with a stronger and more westerly position of the NASH that facilitates the transport of moisture from the Gulf of Mexico, Caribbean Sea, and their adjacent land surfaces to the Mississippi River basin via the Great Plains low-level jet^24, 27, 34^. Our findings suggest that these heavy precipitation events constitute the second phase of a two-phase process in the evolution of a flood; the first phase begins up to year prior to peak discharge, when atmospheric processes connected to El Niño increase surface water storage of the lower Mississippi basin and precondition the basin for enhanced runoff during subsequent precipitation events.

**Figure 4:**
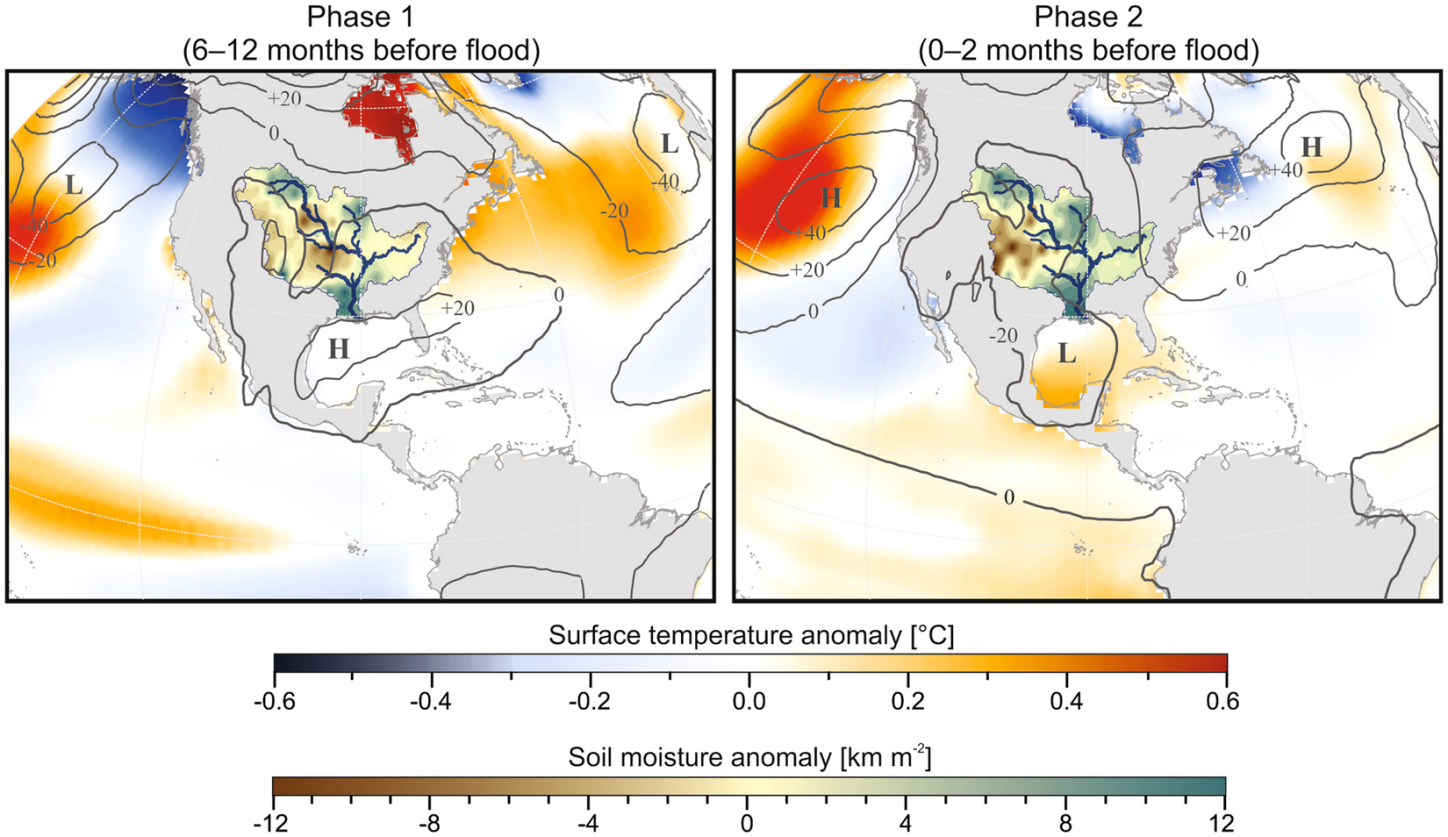
Composited and normalized anomalies of surface temperature over the ocean, sea level pressure (contours), and soil moisture over the Mississippi River basin at 6–12 months (Phase 1) and 0–2 months (Phase 2) prior to simulated extreme floods (n = 116). Plotted variables represent average anomalies of all months in a phase. Sea level pressure anomaly contour intervals are 20 Pa. Maps generated in ArcMap v.10.2.2 (http://arcgis.com).

To further explore the climatological evolution of extreme floods on the lower Mississippi River, we examined modeled ocean-atmosphere dynamics over the western hemisphere and soil moisture anomalies over the Mississippi River basin in the year leading up to simulated extreme floods (Fig. 4). At 6–12 months prior to peak discharge (Phase 1), composite surface temperature anomalies exhibit a pronounced El Niño-like pattern across the cold tongue region in the eastern equatorial Pacific together with persistent low-pressure anomalies over the northern Pacific and Atlantic (Fig. 4a). The climatological conditions preceding flood events resemble the surface temperature and sea level pressure anomalies associated with El Niño events^26, 28^, and are associated with positive soil moisture anomalies over the lower Mississippi basin that increase through to the time of peak discharge. At 0–2 months prior to peak discharge (Phase 2), high-pressure anomalies persist over the North Pacific and Atlantic accompanied by low pressure over central North America (Fig. 4b). This atmospheric configuration mirrors the negative PNA phase that can trigger large floods along the lower Mississippi River and its major tributaries via heavy precipitation in the weeks prior to peak discharge^16, 22–25^. Our analysis demonstrates that these rainstorms are more likely to result in a high magnitude flood if they are preceded by an El Niño event in the previous year, adding substantial predictive capability to forecasts of flood risk.

**Figure 5:**
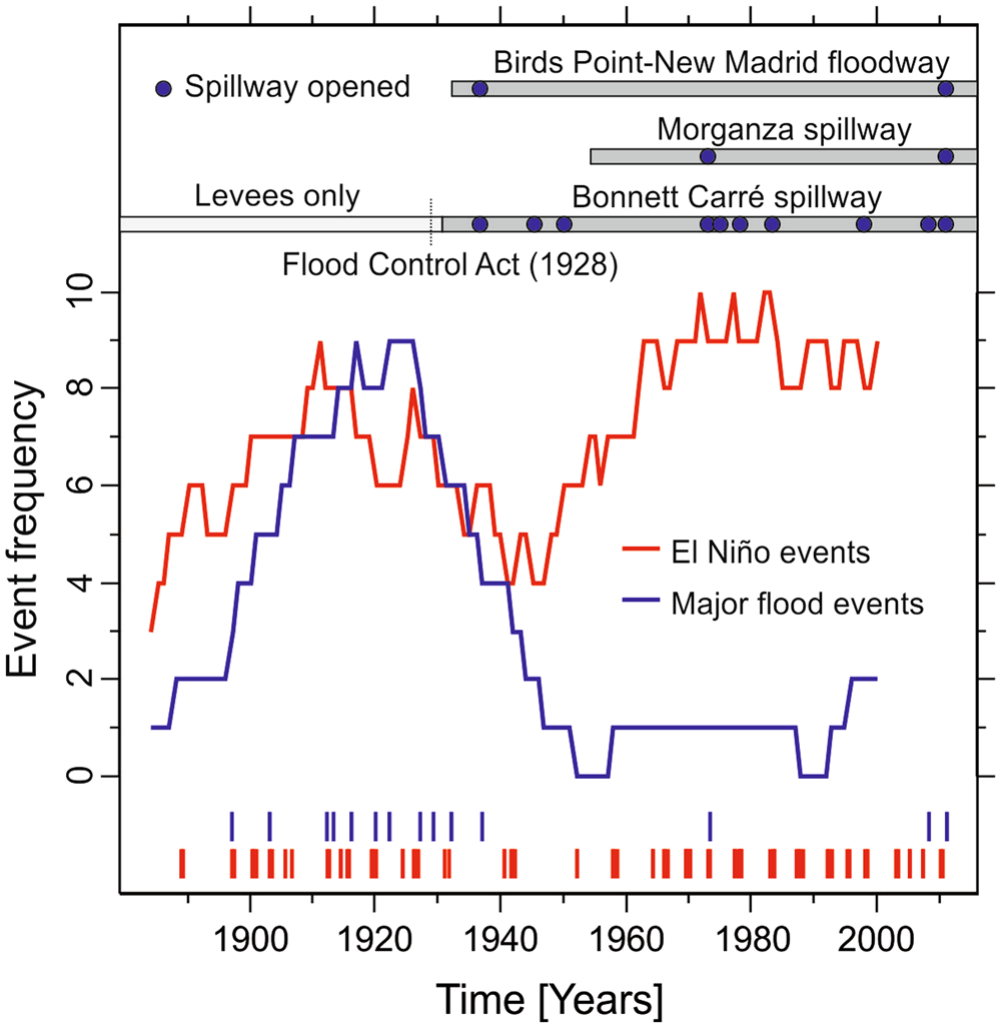
The frequency of historical El Niño events (red) and major Mississippi River floods at Vicksburg, Mississippi (blue) since the late 19^th^ century to present in relation to river engineering along the lower Mississippi River. Event frequencies calculated as the number of events within a moving 30-year window.

Our findings – implying that ENSO variability plays an important role in the development of floods on the lower Mississippi River – are generally consistent with historical observations (Fig. 5). Prior to the mid-20^th^ century when flood control consisted mainly of artificial levees along the main channel, the frequency of major floods at Vicksburg, Mississippi closely tracks the frequency of El Niño events. This relationship has apparently broken down since the mid-20^th^ century, when only three floods – in 1973, 2008, and 2011 – have attained major flood stage despite an increase in the frequency of El Niño events at this time^35^. The timing of this shift in the relationship between flood stages and ENSO variability follows the establishment of the lower Mississippi’s modern flood control system as well as an intensification of other anthropogenic changes to the land surface and hydrology of the Mississippi River basin^2, 6^ that are not included in the CESM-LME simulations. The modern flood control system includes an artificially shortened and straightened main channel held in place by concrete revetments, and a series of spillway structures that can be opened during times of high discharge to relieve pressure on levees that together have altered the relationship between river stage and discharge during the 20^th^ century^2, 6, 7^. The spillway structures have, however, been opened more often during periods of increased El Niño event frequency (e.g., 1970–1985), indicating that ENSO continues to shape inter-annual variability of the lower Mississippi’s discharge despite the strong influence of human activities on the river’s recent behavior.

Our results represent a conceptual advance for near- and long-term forecasts of flood risk for the largest commercial waterway in North America. By augmenting the instrumental record with thousands of years of simulated data contained in the CESM-LME, we identify the two-phase climatological evolution of high-magnitude flooding of the lower Mississippi River, and connect these phases to major modes of climate variability. This analysis provides a consolidated characterization of a typical high-magnitude flood event, but we note the potential for variations on this pattern caused by the potential non-stationarity of ENSO and other modes of internal climate variability^36, 37^, and the sensitivity of hydrological systems to land use and geomorphic processes^38–40^. This work highlights the value of efforts to improve projections of ENSO variability and its influence on surface water resources using the long-term perspective offered by fully coupled model simulations^31–33^ as well as proxy-based reconstructions^25, 41^. If recent projections of increased discharge of the Mississippi River^42^ and changes in the strength and variability of ENSO and its teleconnections under continued greenhouse warming^37, 43, 44^ are correct, our findings imply that anthropogenic climate change increases the risk of extreme flooding on the lower Mississippi River. A shift towards more frequent El Niño events would place additional stress on current flood protection measures and disaster relief services, increasing the likelihood of a historically unprecedented flood capable of undermining existing flood control measures.

## Methods

### Model simulations

We extracted the following variables from all ten CESM–LME full-forcing ensemble members: river discharge (QCHANR) and liquid soil moisture in top 1 m (SOILLIQ) from Community Land Model (CLM), convective precipitation rate (PRECC), large-scale precipitation rate (PRECL), sea level pressure (SLP), surface temperature (TREFHT) from Community Atmosphere Model (CAM), and sea surface temperature (SST) from Community Climate System Model (CCSM). We extracted peak annual river discharge for the lower Mississippi basin (defined as the maximum monthly QCHANR from −92° to −89° longitude, 30° to 37° latitude) for each model year. We also extracted the Niño 3.4 index (defined as the area averaged monthly SST from −170° to −120° longitude and −5° to 5° latitude) for each month, and calculated the Oceanic Niño Index (ONI) as a 3-month running mean of the Niño 3.4 index; El Niño events were defined as periods with 5 consecutive over-lapping months with an ONI > 0.5 °C. We then classified all peak annual discharge values by whether or not they occurred within 12 months of an El Niño event, and calculated recurrence intervals for these discharges using a Log-Pearson Type III distribution^45^.

For each simulated extreme flood (defined as all peak annual discharges with an annual exceedance probability ≤1%; n = 116), we calculated monthly anomalies for SOILLIQ, PRECC, and PRECL (and used the sum of PRECC and PRECL to represent total precipitation, PRECT) for the lower Mississippi basin in the 18 months leading up to and following an extreme flood. We then calculated the mean and 95% confidence intervals of SOILLIQ, PRECT, and the Nino 3.4 index for each month leading up to an extreme flood using the bootstrapping function ‘boot()’ in R v.3.3.0 with 10,000 bootstrap replicates; the same function was used to calculate the 99% confidence intervals for the full series of these variables. To produce maps of SOILLIQ, TREFHT, and SLP, we performed superposed epoch analysis for the extremes in river discharge, and composited normalized anomalies for each field for each month in the year leading up to an extreme flood. We then calculated the mean of these excursions for months 6–12 and 0–2 prior to extreme floods.

### Climate reanalysis data

We extracted monthly volumetric soil moisture at the surface (SOILM) and their long-term monthly means (1981–2010) from the Twentieth Century Reanalysis Project version 2c (V2c)^46^ for the period 1851 to 2014. We used the monthly Niño 3.4 index from ref. 35 to calculate El Niño events in the same way as is described above for the CESM–LME.

### Instrumental river stage and discharge data

We obtained daily and peak annual river stages for the Mississippi River at Vicksburg, Mississippi (Station ID 0728900; −90.902332° longitude, 32.311832° latitude) and Memphis, Tennessee (Station ID 07032000; −90.076670° longitude, 35.123060° latitude) from the United States Geological Survey^29^. Nearly continuous daily flood stages at Vicksburg are available from January 1901 to present, and peak annual stages are available from 1858 to 2015 (sporadically prior to 1903), making this one of the longest river stage records available for the lower Mississippi River; because streamflow along this segment of the river is highly correlated, we used ‘major floods’ (defined as peak annual stages >15.24 m) measured at Vicksburg as representative of floods along the lower Mississippi River.

## supplementary material

Supplementary Material
