# Supplementary material for: El Niño increases the risk of lower Mississippi River flooding
Source: Sci Rep. 2017 May 11;7:1772. doi: 10.1038/s41598-017-01919-6 (PMC5431953; doi:10.1038/s41598-017-01919-6)
Supplement: Supplementary file 1 — Supplementary Material [file 41598_2017_1919_MOESM1_ESM.pdf]

Supplemental Material for 'El Niño increases the risk of lower Mississippi River flooding' by S.E. Munoz & S.G. Dee

Supplemental Table 1. Major floods (peak annual stage > 50 feet; 15.24 m) recorded at the gauging station at Vicksburg, Mississippi (Station ID 0728900) in relation to El Niño events.

Peak stage date		Stage		Flow		Corresponding El Niño[1]			
		ft	m	$ft^3\ s^{-1}$	$m^3\ s^{-1}$	Start date		End date	
1897	April 15	52.48	16.00	1,777,000	50,319	1896	November	1897	March
1903	March 31	51.80	15.79	1,606,000	45,477	1902	September	1903	March
1912	April 12	51.65	15.74	1,780,000	50,404	1912	February	1912	March
1913	May 2	52.20	15.91	1,783,000	50,489	--		--	
1916	February 16	53.85	16.41	1,735,000	49,130	1915	April	1915	July
1920	April 17	50.90	15.51	1,649,000	46,694	1919	January	1919	August
1922	April 20	54.85	16.72	1,752,000	49,611	--		--	
1927	May 1	58.40	17.80	2,278,000	64,506	1925	December	1926	July
1929	June 6	55.10	16.79	1,730,000	48,988	--		--	
1932	February 26	50.07	15.26	1,410,000	39,927	1930	November	1931	June
1937	February 17	52.60	16.03	2,080,000	58,899	--		--	
1973	May 12	50.67	15.44	1,962,000	55,558	1972	October	1973	March
2008	April 20	51.00	15.54	1,820,000	51,537	--		--	
2011	May 17	57.17	17.43	2,310,000	65,412	2009	October	2010	April

[1] Refers to an El Niño event (see methods for definition) that ended within a year prior to major flood stage.

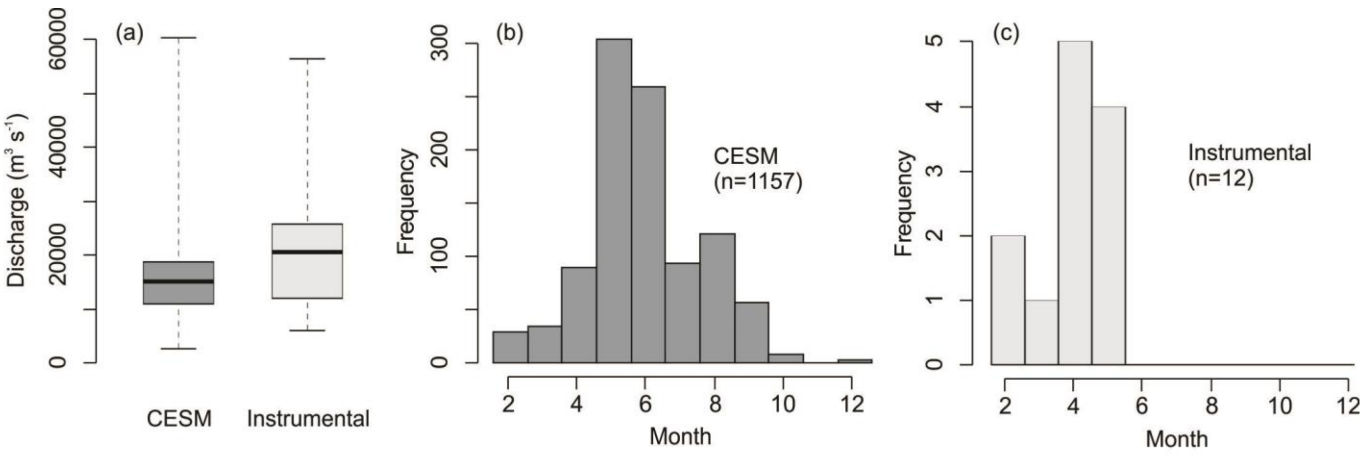

Supplemental Figure 1. Comparison of lower Mississippi River discharge simulated in the CESM–LME with instrumental data from the gauging station at Vicksburg, Mississippi (USGS station no. 7289000); (a) simulated and instrumental (2008-2015) mean monthly discharge, and month of peak annual discharge for the largest 10% of events in (b) simulated and (c) observed (1897-2015) floods.

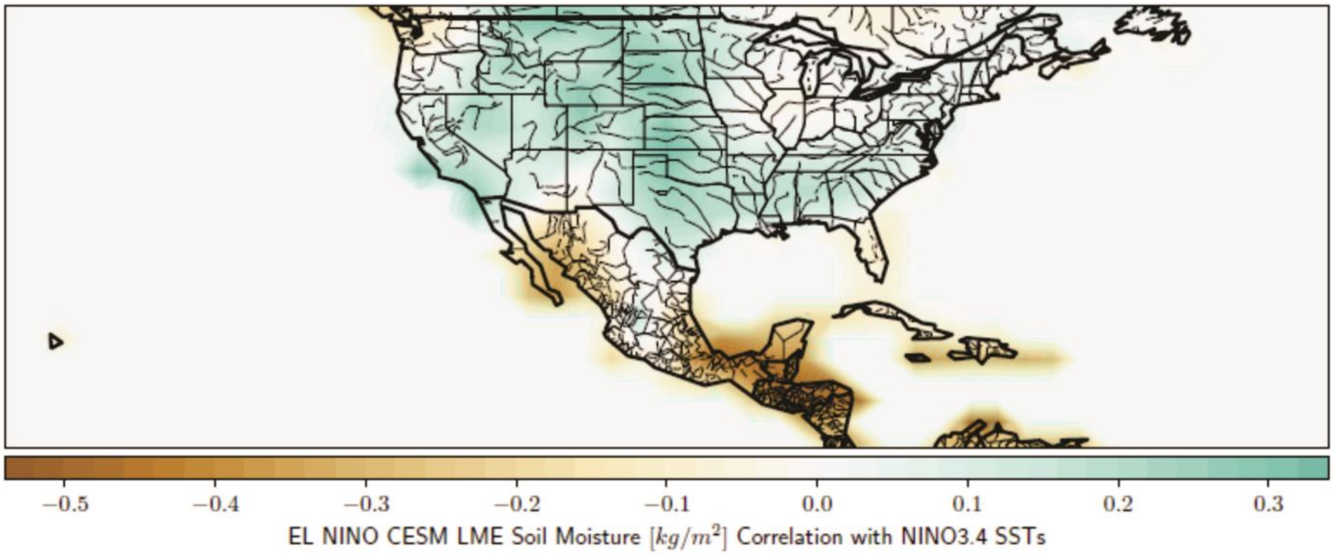

Supplemental Figure 2. Soil moisture in relation to ENSO in the CESM–LME, expressed as a Pearson correlation between monthly soil moisture anomalies and the Niño3.4 index. Patterns simulated in the CESM–LME are comparable to those observed in historical reanalysis data (e.g., Figure 1a), confirming that the CESM provides a suitable depiction of ENSO's teleconnections over the Mississippi River basin. Figure generated in Python 2.7.12 (with packages Basemap, Matplotlib, Numpy, CDMS2).
